# Incidence of idiopathic cardiomyopathy in patients with type 2 diabetes in Taiwan: age, sex, and urbanization status-stratified analysis

**DOI:** 10.1186/s12933-020-01144-y

**Published:** 2020-10-14

**Authors:** Hua-Fen Chen, Ya-Hui Chang, Hsien-Jung Lo, Muhammad Atoillah Isfandiari, Santi Martini, Wen-Hsuan Hou, Chung-Yi Li

**Affiliations:** 1 Department of Endocrinology, Far Eastern Memorial Hospital, New Taipei City, Taiwan; 2 School of Medicine and Department of Public Health, College of Medicine, Fujen Catholic University, New Taipei City, Taiwan; 3 Department of Public Health, College of Medicine, National Cheng Kung University, Tainan, Taiwan; 4 Department of Cardiology, Cardiovascular Center, Far Eastern Memorial Hospital, New Taipei City, Taiwan; 5 Department of Epidemiology, Faculty of Public Health, Universitas Airlangga, Surabaya, Indonesia; 6 Department of Physical Medicine and Rehabilitation, Taipei Medical University Hospital, Taipei, Taiwan; 7 Master Program in Long-Term Care, College of Nursing, Taipei Medical University, Taipei, Taiwan; 8 Graduate Institute of Clinical Medicine, College of Medicine, Taipei Medical University, Taipei, Taiwan; 9 Department of Public Health, College of Public Health, China Medical University, Taichung City, Taiwan; 10 Department of Healthcare Administration, College of Medical and Health Science, Asia University, Taichung City, Taiwan

**Keywords:** Type 2 diabetes mellitus, Cardiomyopathies, Cohort studies, Epidemiology

## Abstract

**Background:**

The epidemiology of diabetes and idiopathic cardiomyopathy have limited data. We investigated the overall and the age-, sex-, and urbanization-specific incidence and relative hazard of idiopathic cardiomyopathy in association with type 2 diabetes and various anti-diabetic medications used in Taiwan.

**Methods:**

A total of 474,268 patients with type 2 diabetes were identified from ambulatory care and inpatient claims in 2007–2009 from Taiwan’s National Health Insurance (NHI) database. We randomly selected 474,266 age-, sex-, and diagnosis date-matched controls from the registry of NHI beneficiaries. All study subjects were linked to ambulatory care and inpatient claims (up to the end of 2016) to identify the possible diagnosis of idiopathic cardiomyopathy. The person-year approach with Poisson assumption was used to estimate the incidence, and Cox proportional hazard regression model with Fine and Gray’s method was used to estimate the relative hazards of idiopathic cardiomyopathy in relation to type 2 diabetes.

**Results:**

The overall incidence of idiopathic cardiomyopathy for men and women patients, respectively, was 3.83 and 2.94 per 10,000 person-years, which were higher than the corresponding men and women controls (2.00 and 1.34 per 10,000 person-years). Compared with the control group, patients with type 2 diabetes were significantly associated with an increased hazard of idiopathic cardiomyopathy (adjusted hazard ratio [aHR]: 1.60, 95% confidence interval [CI]: 1.45–1.77] in all age and sex stratifications except in those men aged > 64 years. Patients with type 2 diabetes aged < 45 years confronted the greatest increase in the hazard of idiopathic cardiomyopathy, with an aHR of 3.35 (95% CI 2.21–5.06) and 3.48 (95% CI 1.60–7.56) for men and women, respectively. The usage of some anti-diabetic medications revealed lower risks of idiopathic cardiomyopathy.

**Conclusions:**

In Taiwan, diabetes increased the risk of idiopathic cardiomyopathy in both sexes and in all age groups, except in men aged > 64 years. Younger patients were vulnerable to have higher HRs of idiopathic cardiomyopathy. Some anti-diabetic medications may reduce the risks of cardiomyopathy.

## Background

Coronary heart disease is the most common cause of cardiovascular complications in diabetes [1]. However, myocardial disorder can still occur in the absence of coronary artery disease, hypertension, valvular disease, and congenital heart disease; thus, this myocardial abnormality might also be associated with diabetes [2]. Diabetic cardiomyopathy is characterized by lipid accumulation in cardiomyocytes, fetal gene reactivation, and left ventricular hypertrophy, which together result in contractile dysfunction [3].

The epidemiology of idiopathic cardiomyopathy in patients with diabetes has not been clear because of the lack of large study outcomes from different diabetic populations. Previous studies were case–control studies [4, 5] or cross-sectional survey [6] with regional hospital- [4], or county [6]-based population rather than the prospective population-based study design. In a cross-sectional study, the prevalence of diabetic cardiomyopathy was reported to be 1.1% in Olmsted County, Minnesota, USA [6]. However, to our best knowledge, no study has yet estimated the incidence of diabetic cardiomyopathy at the population-based level with age and sex stratifications. Some studies [4, 5] did not exclude patients with hypertension diagnosis, which might also have predisposed to cardiomyopathy. In one study, the authors did not exclude ischemic heart disease and valvular heart disease in the control group [5], which might have affected the results of subsequent relative risk estimation. This same study [5] selected the diagnoses of diabetes and cardiomyopathy from the US Nationwide Inpatient Sample, which might have missed some patients with milder symptoms not admitted to the hospital. In addition to the above-mentioned methodological limitations, the urban rural difference in incidence and relative risk of cardiomyopathy in relation to diabetes have not been examined, given that the urban rural difference was observed in some diabetes related complications [7]. We believe that the risks of idiopathic cardiomyopathy related to anti-diabetic medications use have not been evaluated before.

The aim of our study was to use a nationally representative cohort of patients with type 2 diabetes from Taiwan’s National Health Insurance (NHI) claims to investigate the incidence of idiopathic cardiomyopathy in association with type 2 diabetes with particular interest in various age, sex, and urbanization status-stratified analyses. We also assessed the relative hazards of idiopathic cardiomyopathy in association with various anti-diabetic medications.

## Methods

### Study design and subjects

By the end of 1995, approximately 96% of the total Taiwanese population had enrolled in the NHI Program [8], a universal health program implemented by the NHI Administration under the jurisdiction of the Ministry of Health and Welfare. The NHI Administration has had contracted 97% of hospitals and 90% of clinics all over Taiwan [9]. In addition, the NHI Administration performs quarterly expert reviews on a random sample for every 50 to 100 ambulatory and inpatient claims to ensure the accuracy of claim files so that information available is considered to be complete and accurate [10]. We used the data of ambulatory care claims (2006–2016), inpatient claims (2006–2016), registry for beneficiaries (2007–2009), and death certificate registry (2007–2016) for this study. The ambulatory care claims record all outpatient (including emergency room visit)-related information, including personal identification number (PIN), date of birth, sex, and date of outpatient visit with a maximum of three leading diagnostic codes. The inpatient claims include all hospitalization information including PIN, date of birth, sex, and dates of admission and discharge, with a maximum of five leading discharge diagnostic codes and four operation procedure codes. All the dataset can be inter-linked through PIN. The study proposal was approved by the Institutional Review Board of National Cheng Kung University Hospital (A-EX-104-008).

**Fig. 1:**
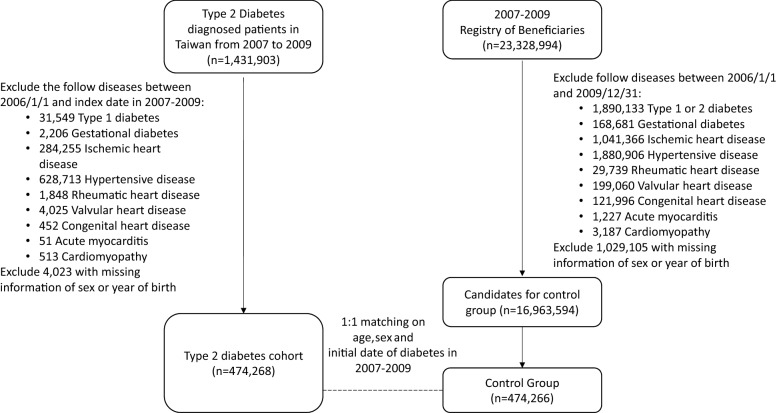
Flowchart for selection of type 2 diabetes cohort and control group

An individual was classified as a type 2 diabetic patient if he or she had an initial type 2 diabetes diagnosis (ICD-9-CM 250. × 0, ICD-9-CM 250. × 2 or ICD-10-CM E11) in ambulatory care and inpatient claims between 2007 and 2009 and then experienced another one or more diagnoses within the subsequent 12 months. Additionally, the first and last outpatient visits during the 12-month period had to be separated by at least 30 days to avoid the accidental inclusion of miscoded patients. The initial diabetic cohort consisted of 1,431,903 patients. We excluded 4,023 subjects with missing information of sex or year of birth, 31,549 patients with type 1 diabetes, and 2,206 patients with gestational diabetes diagnosis between 1 January 2006 and the date of first type 2 diabetes diagnosis in 2007–2009 (i.e., the index date). We also excluded some patients recorded with cardiovascular risk factors for cardiomyopathy in ambulatory care or inpatient claims before the index date. We further excluded 284,255 patients with prior histories of ischemic heart disease, 628,713 patients with prior histories of hypertensive disease, 1848 patients with prior histories of rheumatic heart disease, 4025 patients with prior histories of valvular heart disease, 452 patients with prior histories of congenital heart disease, 51 patients with prior histories of acute myocarditis and 513 patients with prior histories of cardiomyopathy. The final diabetic cohort consisted of 474,268 patients (Fig. 1). Respective ICD-9 and ICD-10 codes are shown in Table 1.

**Table 1:** ICD codes for the diseases analyzed in this study

Diseases	ICD-9-CM	ICD-10-CM
Type 2 diabetes	250. × 0, 250. × 2	E11
Type 1 diabetes	250. × 1, 250. × 3	E10
Gestational diabetes	648.xx	O24.4
Comorbidities		
Ischemic heart disease	410–414	I20-I25
Hypertensive disease	401–405	I10-I16
Rheumatic heart disease	390, 391, 394–398	I00, I01, I05-I09
Valvular heart disease	424	I34-I39
Congenital heart disease	745–747	Q20-Q28
Acute myocarditis	422	I40, I41
Cardiomyopathy	425	I42, I43
Stroke	431–438	I61-I69
Hyperlipidemia	272.0–272.4	E78.0-E78.4
Obesity	278.0	E66
End-point		
Other primary cardiomyopathies	425.4	
Dilated cardiomyopathy		I42.0

*ICD-9-CM* International Classifications of Diseases, Ninth Revision Clinical Modification, *ICD-10-CM* International Classifications of Diseases, Tenth Revision Clinical Modification,

Our control group was collected from the registry of beneficiaries, which contains information, such as PIN, date of birth, sex, geographic area of each member's NHI unit, and dates of enrollment and withdrawal from NHI each time. The registry of beneficiaries enrolled 23,328,994 individuals between 2007 and 2009. We excluded 1,029,105 subjects with missing information of sex or year of birth, 1,890,133 patients with type 1 or type 2 diabetes, 168,681 patients with gestational diabetes, 1,041,366 patients with prior histories of ischemic heart disease, 1,880,906 patients with prior histories of hypertensive disease, 29,739 patients with prior histories of rheumatic heart disease, 199,060 patients with prior histories of valvular heart disease, 121,996 patients with prior histories of congenital heart disease, 1227 patients with prior histories of acute myocarditis, and 3187 patients with prior histories of cardiomyopathy recorded in either ambulatory care or inpatient claims between 1 January 2006 and the index date (Fig. 1).

We used the individual matching technique for control selection. We randomly selected one control by matching one patient with type 2 diabetes on age, sex, and the index date of type 2 diabetes diagnosis. A total of 474,266 controls were selected from the 16,963,594 potential controls. The index date for subjects in the control group was the same as his/her matched type 2 diabetes.

The difference in time between the index date and the date of birth were set as the age of each study subject. We grouped the township/city of each member's NHI unit, either the beneficiaries' residential area or the location of their employment, into two urbanization statuses (urban and rural) according to the classification scheme by Liu et al. [11].

### Follow-up, study end-points, and covariate

We linked the study subjects to ambulatory and inpatient claims from the index date to the last day of 2016 using their unique PINs to identify the primary or secondary diagnostic codes of the following idiopathic cardiomyopathy diagnoses as the end point of this study: other primary cardiomyopathies (ICD-9-CM: 425.4) or dilated cardiomyopathy (ICD-10-CM: I42.0). The minimal criteria to diagnose diabetic cardiomyopathy include left ventricular diastolic dysfunction and/ or reduced left ventricular ejection fraction, pathological left ventricular hypertrophy, and interstitial fibrosis [12], which might be easily identified by non-invasive echocardiogram after clinically ruling out other acquired disorders such as ischemic, hypertensive, rheumatic, valvular and congenital heart diseases. However, the NHI claims provided by the Ministry of Health and Welfare included no information about detailed medical records and investigation results.

Each study subject was followed from the index date to the date of idiopathic cardiomyopathy diagnosis, death censoring, or the last day of 2016, whichever came first. Information on various cardiovascular risk factors for cardiomyopathy including ischemic heart disease, hypertensive disease, rheumatic heart disease, valvular heart disease, congenital heart disease, acute myocarditis, stroke, obesity, and hyperlipidemia, were retrieved from ambulatory care and inpatient claims between index date and date of end-of-follow-up and considered as potential confounders.

We also collected the information of antidiabetic medications (sulphonylureas [SU], meglitinides, thiazolidinediones [TZD], α-glucosidase inhibitors [AGi], metformin, dipeptidyl peptidase 4 inhibitors [DPP-4i], sodium glucose cotransporter 2 inhibitors [SGLT-2i], insulin, and glucagon-like peptide 1 receptor agonists [GLP-1]) and antihypertensive medications (β-blockers, angiotensin converting enzyme inhibitors [ACEi], and angiotensin receptor blockers [ARB]) and evaluated their effects on the risk of idiopathic cardiomyopathy. Information about antidiabetic and antihypertensive medications was retrieved between the index date and the end of follow-up.

### Statistical analysis

The age- and sex-specific incidence density estimates were calculated with person-years as the denominator under the Poisson assumption. We assessed the independent association of type 2 diabetes with the risk cardiomyopathy by conducting Cox proportional hazard regression model with Fine and Gray’s method to account for “death” as a potential competing risk event. The model was also adjusted for age, sex, urbanization status, cardiovascular risk factors, and antihypertensive medications use. Adjustment for the geographic variables may help reduce the presence of an urban rural difference in accessibility to medical health services in Taiwan [13]. We adjusted cardiovascular risk factors that occurred after baseline type 2 diabetes, which might result in a potential over-adjustment of these comorbidities, as some of these cardiovascular risk factors could play a role of mediator located on the causal pathway from type 2 diabetes to cardiomyopathy. To address this potential problem, we conducted a sensitivity analysis that removed adjustment for these confounders.

We also evaluated the relative hazards of idiopathic cardiomyopathy according to diabetic status, age, sex, urbanization status, various comorbidities, and antihypertensive medication use. Based on the sample of type 2 diabetes only, we also assessed the associations of selected anti-diabetic medications with idiopathic cardiomyopathy. To address the contributions of type 2 diabetes and controls with various cardiovascular risk factors to the risk of idiopathic cardiomyopathy, we performed Cox proportional hazard regression model to assess the risk of idiopathic cardiomyopathy in relation to type 2 diabetes and cardiovascular risk factors.

All statistical analyses and survival curves were performed with SAS (version 9.4; SAS Institute, Cary, NC). A p value < 0.05 was considered statistically significant.

## Results

The mean age ± standard deviation (SD) of the type 2 diabetic and control group was similar at 55.84 ± 13.20 years, and both groups were male predominant. The urban rural differences for the two groups were also comparable. Patients with type 2 diabetes tended to have higher prevalence of ischemic heart disease, hypertensive disease, rheumatic, valvular, congenital heart diseases, acute myocarditis, stroke, obesity and hyperlipidemia after the index date. The median time of follow-up was 9.15 ± 1.36 years and 8.91 ± 1.68 years in the control and type 2 diabetes groups, respectively (Table 2).

**Table 2:** Characteristics of the study subjects

Variables ^a^	Control group		Diabetic group		p value
	*n*	%	*n*	%	
General characteristics					
Age					
< 45	85,872	18.11	85,872	18.11	1.0000
45–64	268,869	56.69	268,869	56.69	
> 64	119,525	25.20	119,527	25.20	
Mean age (± SD)	55.84	13.20	55.84	13.20	0.9912
Sex					
Male	267,161	56.33	267,163	56.33	0.9986
Female	207,105	43.67	207,105	43.67	
Urbanization status					
Urban area	342,117	72.14	332,301	70.07	< 0.0001
Rural area	132,144	27.86	141,963	29.93	
Follow-up period (year) (± SD)	9.15	1.36	8.91	1.68	< 0.0001
Comorbidities					
Ischemic heart disease	55,224	11.64	104,857	22.11	< 0.0001
Hypertensive disease	141,639	29.86	267,425	56.39	< 0.0001
Rheumatic heart disease	4413	0.93	5466	1.15	< 0.0001
Valvular heart disease	17,717	3.74	18,464	3.89	< 0.0001
Congenital heart disease	1398	0.29	1901	0.40	< 0.0001
Acute myocarditis	62	0.01	114	0.02	< 0.0001
Stroke	54,290	11.45	108,520	22.88	< 0.0001
Obesity	3432	0.72	14,139	2.98	< 0.0001
Hyperlipidemia	133,807	28.21	351,046	74.02	< 0.0001
Antihypertensive medication					
β-Blockers	54,974	11.59	112,678	23.76	< 0.0001
ACEi	29,942	6.31	122,133	25.75	< 0.0001
ARB	69,393	14.63	199,158	41.99	< 0.0001
Total	474,266	100.00	474,268	100.00	

*ACEi* angiotensin converting enzyme inhibitors, *ARB* angiotensin receptor blockers

^a^Inconsistency between total population and population summed for individual variable was due to missing information

The overall and age- and sex-specific incidence densities and hazard ratios (HRs) of idiopathic cardiomyopathy are presented in Table 3. The overall incidence density for men and women with type 2 diabetes was 3.83 and 2.94 per 10,000 person-years, respectively, whereas the corresponding figures for men and women in the control group were lower at 2.00 and 1.34 per 10,000 person-years. In both groups, the incidence density of idiopathic cardiomyopathy increased with age except in diabetic men aged 45–64 years, and the highest incidence density was found in the age group > 64 years irrespective of age and diabetic status. Generally, the age- and sex-specific incidence densities of idiopathic cardiomyopathy in patients with type 2 diabetes was higher than those of the control group, but the difference in the incidences in both groups became narrower with increasing age.

**Table 3:** Overall and age- and sex-specific incidence densities and relative hazards of idiopathic cardiomyopathy (ICD9 = 425.4; ICD10 = I42.0) in the diabetic and control groups (Fine and Gray's method)

Variables	Control group				Diabetic group				Crude HR^a^	Adjusted HR^a,e^
	No. of patients	No. of events	Person-years	ID^a^ (per 10,000 patient-years)	No. of patients	No. of events	Person-years	ID^a^ (per 10,000 patient-years)		
Men										
< 45	54,958	40	500,374	0.80	54,958	176	490,078	3.59	4.34 (3.08–6.13)	3.35 (2.21–5.06)^b^
45–64	152,390	253	1,402,959	1.80	152,390	485	1,368,095	3.55	1.93 (1.66–2.25)	1.52 (1.27–1.81)^b^
> 64	59,813	192	522,269	3.68	59,815	241	498,478	4.83	1.26 (1.04–1.52)	1.14 (0.92–1.40)^b^
Total	267,161	485	2,425,602	2.00	267,163	902	2,356,651	3.83	1.86 (1.67–2.08)	1.51 (1.33–1.72)^c^
Women										
< 45	30,914	9	282,840	0.32	30,914	58	280,289	2.07	6.45 (3.2–13.02)	3.48 (1.60–7.56)^b^
45–64	116,479	93	1,088,808	0.85	116,479	276	1,072,278	2.57	2.94 (2.33–3.73)	2.21 (1.67–2.92)^b^
> 64	59,712	155	541,660	2.86	59,712	216	516,964	4.18	1.40 (1.14–1.72)	1.39 (1.11–1.74)^b^
Total	207,105	257	1,913,308	1.34	207,105	550	1,869,531	2.94	2.14 (1.85–2.48)	1.79 (1.52–2.12)^c^
Overall	474,266	742	4,338,910	1.71	474,268	1452	4,226,182	3.44	1.96 (1.79–2.14)	1.60 (1.45–1.77)^d^

^a^Based on Poisson assumption, *ID* incidence density, *HR* hazard ratio

^b^Based on Cox proportional hazard regression with adjustment for urbanization status; status of ischemic heart disease, hypertensive disease, rheumatic heart disease, valvular heart disease, congenital heart disease, acute myocarditis, stroke, obesity, and hyperlipidemia; and antihypertensive medications use

^c^Based on Cox proportional hazard regression with adjustment for age and urbanization status; status of ischemic heart disease, hypertensive disease, rheumatic heart disease, valvular heart disease, congenital heart disease, acute myocarditis, stroke, obesity, and hyperlipidemia; and antihypertensive medications use

^d^Based on Cox proportional hazard regression with adjustment for age, sex and urbanization status; status of ischemic heart disease, hypertensive disease, rheumatic heart disease, valvular heart disease, congenital heart disease, acute myocarditis, stroke, obesity, and hyperlipidemia; and antihypertensive medications use

^e^p values for the interaction of diabetes and sex, diabetes and age in males, and diabetes and age in females were 0.2088, < 0.0001, and < 0.0001, respectively

Men and women with type 2 diabetes were observed to experience increased hazard of idiopathic cardiomyopathy with crude HRs of 1.86 (95% confidence interval [CI] 1.67–2.08) and 2.14 (95% CI 1.85–2.48), respectively. Further adjustment for age, sex, urbanization status, and cardiovascular risk factors attenuated the HRs to 1.51 (95% CI 1.33–1.72) and 1.79 (95% CI 1.52–2.12) in male and female with type 2 diabetes, respectively. Because of a significant interaction of type 2 diabetes status with age (p < 0.0001) in men and women, we performed stratified analysis to estimate the age-specific HR for each sex. Patients with type 2 diabetes patients aged < 45 years had the highest adjusted hazard ratios (aHRs: 3.35 [95% CI 2.21–5.06] in men and 3.48 [95% CI 1.60–7.56] in women). The HRs attenuated with increasing age and it became inconsequential after adjustment for covariates (Table 3) in those over aged 64 years old for men but not in women.

We performed stratified analyses to estimate the urbanization status-specific HRs for each sex. (Table 4). A higher incidence of idiopathic cardiomyopathy was observed in men and women from the rural areas than in those from the urban areas irrespective of their diabetes status. The aHR of idiopathic cardiomyopathy was slightly higher in men in rural areas (HR: 1.55, 95% CI 1.26–1.93) than those in urban areas (HR: 1.49, 95% CI 1.27–1.74). Similarly, the aHRs of cardiomyopathy was higher in women from rural areas than their urban counterparts (HR 2.03 vs. 1.63).

**Table 4:** Overall and urbanization- and sex-specific incidence densities and relative hazards of idiopathic cardiomyopathy (ICD9 = 425.4; ICD10 = I42.0) in the diabetic and control groups. (Fine and Gray's method)

Variables^a^	Control group				Diabetic group				Crude HR^b^	Adjusted HR^b,f^
	No. of patients	No. of events	Person-years	ID^b^ (per 10,000 patient-years)	No. of patients	No. of events	Person-years	ID^b^ (per 10,000 patient-years)		
Men										
Urban	190,969	325	1,737,479	1.87	187,691	591	1,661,658	3.56	1.86 (1.62–2.13)	1.49 (1.27–1.74)^c^
Rural	76,189	160	688,093	2.33	79,468	311	694,967	4.48	1.86 (1.54–2.25)	1.55 (1.26–1.93)^c^
Total	267,161	485	2,425,602	2.00	267,163	902	2,356,651	3.83	1.86 (1.67–2.08)	1.51 (1.33–1.72)^d^
Women										
Urban	151,148	164	1,397,475	1.17	144,610	325	1,307,333	2.49	2.08 (1.72–2.51)	1.63 (1.32–2.02)^c^
Rural	55,955	93	515,813	1.80	62,495	225	562,198	4.00	2.15 (1.69–2.74)	2.03 (1.55–2.66)^c^
Total	207,105	257	1,913,308	1.34	207,105	550	1,869,531	2.94	2.14 (1.85–2.48)	1.79 (1.52–2.12)^d^
Overall	474,266	742	4,338,910	1.71	474,268	1452	4,226,182	3.44	1.96 (1.79–2.14)	1.60 (1.45–1.77)^e^

^a^Inconsistency in the total numbers of patients and person-years between the total population and those summed for population from urban and rural areas was due to missing information of urbanization status for some study subjects

^b^Based on Poisson assumption, *ID* incidence density, *HR* hazard ratio

^c^Based on Cox proportional hazard regression with adjustment for age; status of ischemic heart disease, hypertensive disease, rheumatic heart disease, valvular heart disease, congenital heart disease, acute myocarditis, stroke, obesity, and hyperlipidemia; and antihypertensive medications use

^d^Based on Cox proportional hazard regression with adjustment for age and urbanization status; status of ischemic heart disease, hypertensive disease, rheumatic heart disease, valvular heart disease, congenital heart disease, acute myocarditis, stroke, obesity, and hyperlipidemia; and antihypertensive medications use

^e^Based on Cox proportional hazard regression with adjustment for age, sex, and urbanization status; status of ischemic heart disease, hypertensive disease, rheumatic heart disease, valvular heart disease, congenital heart disease, acute myocarditis, stroke, obesity, and hyperlipidemia; and antihypertensive medications use

^f^p value for the interaction of diabetes and sex, diabetes and urbanization in males, and diabetes and urbanization in females were 0.2088, 0.8646, and 0.6166, respectively

The relative hazards of idiopathic cardiomyopathy in relation to type 2 diabetes status, ages, sex, urbanization status, various cardiovascular risk factors, and antihypertensive medications use are presented in Table 5. Type 2 diabetes significantly increased the risk of idiopathic cardiomyopathy (HR: 1.60, 95% CI 1.45–1.77) after the adjustment of potential confounders. Male sex, age > 64 years, living in rural areas, having a history of ischemic, rheumatic, valvular, congenital heart diseases, or acute myocarditis, and taking antihypertensive medications (β-blockers, ACEi, or ARB) increased the risk of idiopathic cardiomyopathy in both crude and adjusted analyses. However, the increased risks of those aged 45–64 years and with a history of hypertension, stroke, or hyperlipidemia were not sustained after the adjustment of covariates. Obesity was not related to idiopathic cardiomyopathy in crude and adjusted analyses. The different relative hazards of the control subjects and patients with type 2 diabetes with various cardiovascular risk factors can be found in Additional file 1: Table S1.

**Table 5:** The relative hazards of idiopathic cardiomyopathy (ICD9 = 425.4; ICD10 = I42.0) in the diabetic and control groups (Fine and Gray's method)

	Crude HR	Adjusted HR^a^
General characteristics		
Type 2 diabetes	1.96 (1.79–2.14)	1.60 (1.45–1.77)
Age		
< 45	1.0	1.0
45–64	1.23 (1.08–1.41)	0.99 (0.87–1.14)
> 64	2.03 (1.77–2.32)	1.32 (1.13–1.53)
Sex		
Female	1.0	1.0
Male	1.34 (1.23–1.46)	1.42 (1.30–1.55)
Urbanization status		
Urban area	1.0	1.0
Rural area	1.38 (1.26–1.5)	1.22 (1.12–1.34)
Comorbidities		
Ischemic heart disease	6.54 (6.01–7.12)	4.60 (4.13–5.12)
Hypertensive disease	2.18 (2–2.37)	0.86 (0.75–0.99)
Rheumatic heart disease	9.02 (7.77–10.47)	2.41 (2.03–2.86)
Valvular heart disease	6.68 (6.03–7.41)	2.94 (2.59–3.34)
Congenital heart disease	4.83 (3.48–6.72)	2.03 (1.45–2.85)
Acute myocarditis	22.75 (11.9–43.48)	7.81 (3.91–15.6)
Obesity	1.12 (0.84–1.51)	1.06 (0.78–1.42)
Stroke	1.43 (1.29–1.58)	0.74 (0.66–0.82)
Hyperlipidemia	1.14 (1.05–1.24)	0.60 (0.55–0.66)
Antihypertensive medication		
β-Blocker	3.34 (3.06–3.63)	1.70 (1.54–1.88)
ACEi	3.47 (3.18–3.78)	2.01 (1.80–2.25)
ARBs	2.43 (2.23–2.64)	1.26 (1.11–1.43)

*ACEi* angiotensin converting enzyme inhibitors, *ARB* angiotensin receptor blockers, *HR* hazard ratio

^a^Based on Cox proportional hazard regression with adjustment for age, sex, and urbanization status; status of ischemic heart disease, hypertensive disease, rheumatic heart disease, valvular heart disease, congenital heart disease, acute myocarditis, stroke, obesity, and hyperlipidemia; and antihypertensive medications use

**Fig. 2:**
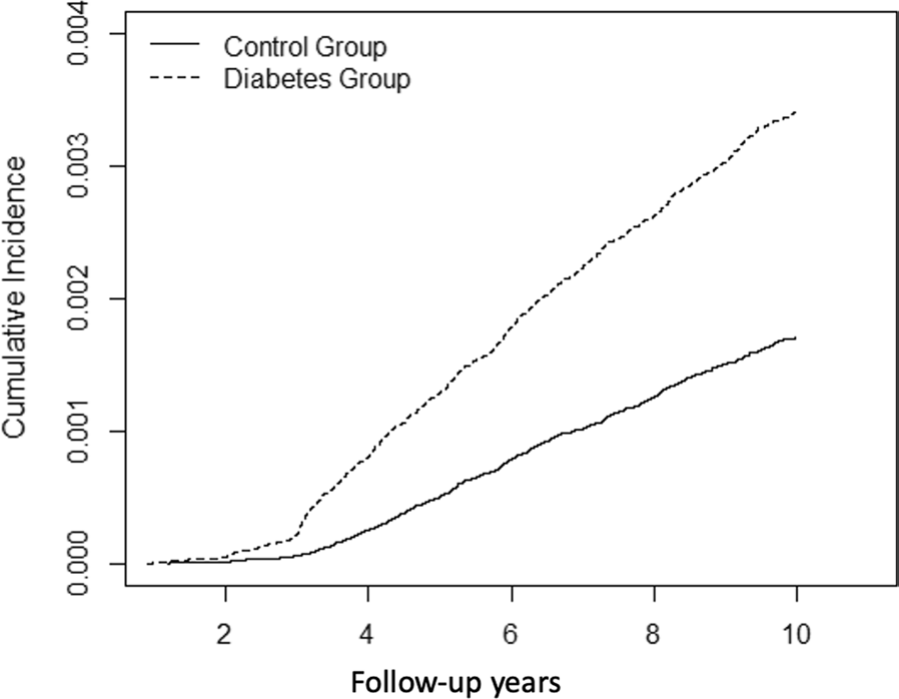
Kaplan–Meier survival curves of the cumulative incidence of idiopathic cardiomyopathy in the diabetic and control groups

Figure 2 presents the Kaplan–Meier survival curves for idiopathic cardiomyopathy in the diabetic and control groups over a 9-year period. The patients with type 2 diabetes were more susceptible to idiopathic cardiomyopathy than the control group with cumulative event rates around 3.06/1000 and 1.56/1000, respectively (p for log-rank test < 0.0001).

Among the patients with type 2 diabetes, those with history of taking SU and AGi did not have an increased risk of idiopathic cardiomyopathy, whereas those with a history of taking metformin, meglitinides, TZD, DPP-4i, SGLT-2i, or GLP-1 had lowered risk. Among the antidiabetic medications, insulin use substantially increased the risk of idiopathic cardiomyopathy (HR: 1.59, 95% CI 1.43–1.78; Table 6).

**Table 6:** Overall relative hazards of idiopathic cardiomyopathy (ICD9 = 425.4; ICD10 = I42.0) in association with anti-diabetic medications use in patients with type 2 diabetes

	Without Cardiomyopathy		With Cardiomyopathy		p value	Total	Adjusted HR^a^
	n	%	n	%			
Sulphonylureas							
No	109,968	23.26	296	20.56	0.0154	110,264	
Yes	362,860	76.74	1144	79.44		364,004	0.98 (0.86–1.13)
Meglitinides							
No	363,996	76.98	1077	74.79	0.0486	365,073	
Yes	108,832	23.02	363	25.21		109,195	0.85 (0.75–0.95)
Thiazolidinediones							
No	316,148	66.86	977	67.85	0.4283	317,125	
Yes	156,680	33.14	463	32.15		157,143	0.83 (0.74–0.93)
α-Glucosidase inhibitors							
No	314,316	66.48	922	64.03	0.0495	315,238	
Yes	158,512	33.52	518	35.97		159,030	0.89 (0.80–1.00)
Metformin							
No	85,663	18.12	281	19.51	0.1695	85,944	
Yes	387,165	81.88	1159	80.49		388,324	0.80 (0.70–0.92)
Insulin							
No	350,379	74.10	824	57.22	< .0001	351,203	
Yes	122,449	25.90	616	42.78		123,065	1.59 (1.43–1.78)
Dipeptidyl peptidase 4 inhibitors							
No	257,876	54.54	929	64.51	< .0001	258,805	
Yes	214,952	45.46	511	35.49		215,463	0.50 (0.45–0.56)
Sodium glucose cotransporter 2 inhibitors							
No	461,571	97.62	1438	99.86	< .0001	463,009	
Yes	11,257	2.38	2	0.14		11,259	0.06 (0.02–0.24)
Glucagon-like peptide 1 receptor agonists							
No	469,889	99.38	1438	99.86	0.0198	471,327	
Yes	2939	0.62	2	0.14		2941	0.23 (0.06–0.91)

*HR* hazard ratio

^a^Based on Cox proportional hazard regression with adjustment for age, sex, and urbanization status; status of ischemic heart disease, hypertensive disease, rheumatic heart disease, valvular heart disease, congenital heart disease, acute myocarditis, stroke, obesity, and hyperlipidemia; and antihypertensive medications use

## Discussion

In our study, the overall incidence densities of idiopathic cardiomyopathy in Taiwan were higher in patients with type 2 diabetes than in controls. The incidence increased with age, and those aged > 64 had the highest incidence in both groups. Furthermore, men tended to have higher incidence rate than women regardless of diabetic status. Our data also demonstrated that diabetes increased the risk of idiopathic cardiomyopathy, and those aged < 45 had the highest risk. The relative risk attenuated with increasing age, and it became unremarkable in those men aged > 64 years.

The incidence estimates of idiopathic cardiomyopathy in the control group in Taiwan was lower than that of previous general population-based study from Minnesota, USA [14], higher than that of western Denmark [15], and comparable to that of a Qatar study between 1996 and 2002 [16]. Direct comparisons of the incidence densities of idiopathic cardiomyopathy between ours and that of previous general population-based studies might be inappropriate because of dissimilarity in baseline demographic status, variations in methods of outcome ascertainment, and length of follow-up, but ethnicity-specific variations in cardiac structure and function might have contributed to difference in incidence estimates in various countries. Black and Hispanic patients with diabetic cardiomyopathy have greater left ventricular mass and wall thickness, but neither White nor Chinese patients with diabetic cardiomyopathy have increased left ventricular mass after adjustment for demographic and anthropomorphic factors in the Multi-Ethnic Study of Atherosclerosis study [17]. Diabetes is strongly associated with lower end diastolic volume among Whites; the association is more modest among Chinese and Blacks and is not present in Hispanics, whereas stroke volume is considerably lower in Whites, Chinese, and Blacks with diabetes [17]. In another study, European patients with type 2 diabetic cardiomyopathy have higher myocardial triglyceride concentration whereas their Asian counterparts have a higher left ventricular mass with lower extracellular volume fraction. The increased left ventricular concentricity in the diabetic cardiomyopathy of the European patients was due to reduction in left ventricular end diastolic volume, whereas that in the Asian patients was due to increase in left ventricular mass [18].

Few population studies discussed the incidence of idiopathic cardiomyopathy in different sexes. In Olmsted County, Minnesota, USA [14], the total incidence of idiopathic cardiomyopathy in men and women are 7.6 and 2.5 per 10^5^ person-years, which are higher than those of the control group in Taiwan (2.00 and 1.34). In their report [14], men have higher incidence than women in all age groups, which is similar to our results. Sex and gender differences in genetics, pathophysiology, immune system, myocardial inflammation, and cardiac remodeling [19], together with different prevalence rate of diabetes in various race and ethnicities [20], might have been responsible for such discrepancy in the results of the previous study and ours. Androgens promote cardiac hypertrophy, and men develop atherosclerotic plaques earlier and more extensively than women; estrogen in women prevents apoptosis in cardiac myocytes, inhibits reactive oxygen species-induced cardiac damage, and opposes mechanisms that lead to cardiac hypertrophy and fibrosis [19].

To the best of knowledge, this is the first population-based study that evaluated the incidence of idiopathic cardiomyopathy in patients with diabetes. In Olmsted County, Minnesota, USA, the authors estimated that the prevalence of diabetic cardiomyopathy is 1.1% in community population and 16.9% in patients with diabetes [6]. The annual hospital discharge rate of idiopathic cardiomyopathy in the Nationwide Inpatient Sample was ascertained to be quite high at around 76 per 10,000 patients with diabetes in the USA [5]. The inclusion of patients with hypertension, which is also a predisposing factor of cardiomyopathy, as well as the inability to identify the multiple hospitalizations of the same individual in their study, might have overestimated the discharge rate of cardiomyopathy.

In Taiwan, the crude HR of idiopathic cardiomyopathy in patients with diabetes (HR: 1.96) was slightly higher than the univariate odds ratio (OR) of idiopathic cardiomyopathy in Bertoni et al.’s study (1.75) [5], but lower than the OR of Coughlin’s Washington DC Dilated Cardiomyopathy Study in the USA (2.6) [4]. Bertoni and our studies used ICD codes for outcome ascertainment, whereas Coughlin et al. restricted their cases to echocardiographic evidence of regional wall motion abnormality, ventricular dilatation and hypokinesia. Such difference in outcome definition might have affected the results. After adjustment of related risk factors, the aHR of our estimates (1.60) was comparable with the adjusted OR (1.58) of Bertoni et al.’s study [5]. The HR became insubstantial in men aged > 64 years in our study. Two previous case–control studies that recruited subjects older than 60 years of age also found out that the association of idiopathic dilated cardiomyopathy with diabetes is of borderline significance (p < 0.10) [21]. The insubstantial association between diabetes and idiopathic cardiomyopathy in the elderly Taiwanese population may have highlighted a greater association between age and cardiomyopathy. In addition, subsequent sensitivity analysis in our study showed that the HRs of idiopathic cardiomyopathy only slightly elevated. Thus, the cardiovascular comorbidities did not meaningfully mediate the association between diabetes and idiopathic cardiomyopathy.

The relative risk carried by type 2 diabetes is greater in women at any age stratum, which was independent of rural or urban status. Actually, the incidence of idiopathic cardiomyopathy in females in the control group was very low compared to that of males in the control group in all age groups. Similar findings of the low incidence of other cardiovascular complications in the female control group had been discussed in our previous reports [22, 23]. The absolute rates of cardiovascular disease among individuals without diabetes are higher in men than in women at all ages [24]. However, more severe endothelial dysfunction and abnormal fibrinolysis [25] found in diabetic women weaken the cardioprotection that is considered to occur in premenopausal women. Women with type 2 diabetes are more likely to be obese [26], hypertensive [27], less physically active [24] and have hypercholesterolemia [27], but are less likely to be prescribed optimal therapy than their male counterparts [27, 28].

The pathophysiological mechanisms of diabetic cardiomyopathy have not been clearly elucidated. The oxidative stress induced by hyperglycemia leads to reduced myocardial contractility and fibrosis [29]. Insulin resistance and subsequent hyperinsulinemia and lipotoxicity [30] are associated with the increased incidence and progression of coronary artery calcification [31], structural and morphological alterations, and impaired myocardial performance. Endoplasmic reticulum stress, impaired calcium handling, mitochondrial dysfunction, autophagy, posttranslational modification, microRNAs modulation [30, 32], DNA methylation, histone modifications [30], and inflammatory cytokine-mediated alterations in vascular function and structure [33] are also associated with the pathogenesis of diabetic cardiomyopathy. In addition, myocardial fibrosis, coronary microcirculation alternations, smooth muscle cell dysfunction, extramural compression, luminal obstruction, and vascular remodeling are also related to cardiomyopathy [34, 35].

Urban–rural differences in the incidence and relative risk of idiopathic cardiomyopathy in patients with diabetes were rarely discussed before. Patients from rural areas in Taiwan are older and have more chronic diseases than their urban and suburban counterparts [36], but patients with diabetes who live in rural areas are less likely to receive guideline-recommended examinations or tests [37]. Although the universal health insurance has largely removed financial barriers to health care, the urban–rural disparity in prevalence of diabetic complications still exists after nearly two decades of implementation of the NHI program in Taiwan [38]. Further studies are necessary to detect the definite underlying etiologies and measures to eliminate such urban–rural difference in various diabetic complications, including idiopathic cardiomyopathy.

Although hypertension is an important risk factor of cardiomyopathy, hypertension did not increase the HR of idiopathic cardiomyopathy after the adjustment of confounding factors in our analysis. Excluding cases of hypertrophic cardiomyopathy, the more common type of hypertension-associated cardiomyopathy [39], could attribute to these results. Overweight/obese individuals with type 2 diabetes have a higher prevalence and odds of left ventricular hypertrophy and diastolic dysfunction [40]. Thus, obesity is hypothesized as a predisposing factor for diabetic cardiomyopathy. In our study, however, obesity did not increase the risk of idiopathic cardiomyopathy before and after the adjustment of risk factors. Stroke and hyperlipidemia, which are the common comorbidities of type 2 diabetes, were not associated with increased risk of idiopathic cardiomyopathy after the adjustment of other cardiovascular risk factors.

ACEi reduces the risk of new onset heart failure in patients with established cardiovascular disease or diabetes mellitus, and ARB improves calcium signaling parameters in atrial tissue with diabetic cardiomyopathy [41]. However, the use of β-blockers in patients with diabetes mellitus is associated with an increased risk for cardiovascular events [42]. In our study, the usage of β-blockers, ACEi and ARB increased the risk of idiopathic cardiomyopathy even after the adjustment of other cardiovascular variables. ACEi, ARB, and β-blockers are guideline-recommended pharmacotherapy for heart failure, which is the most common complication of cardiomyopathy [43]. Whether the increased risk of idiopathic cardiomyopathy observed in people with such antihypertensive medications was indicated by heart failure deserves further research.

This study might be the first one to evaluate the risks of idiopathic cardiomyopathy in association with various anti-diabetic medications use. In our study, insulin increased the risk of idiopathic cardiomyopathy. Insulin-treated patients with diabetes are likely to be older, have longer duration of diabetes, and have more comorbidities, including atherosclerotic disease [44]. Thus, the causal relationship between insulin therapy and the risk of idiopathic cardiomyopathy should be interpreted with caution. Previous randomized controlled trial [45] and a retrospective cohort study [46] did not find a relationship between insulin therapy and adverse cardiovascular outcome; hence, further research is mandatory to confirm or refute such association.

Metformin, the first-line recommended anti-diabetic medication, was associated with lower risk of idiopathic cardiomyopathy. In previous studies, metformin has reduced the risk of myocardial infarction [47], and mortality in patients with diabetes and heart failure [48]. Metformin improves vascular endothelial function, hemostasis and glycoxidation, and exerts cellular antiatherogenic effects [49], which might be responsible for the reduced risk of idiopathic cardiomyopathy.

Although the PROactive [50] and RECORD [51] trials showed a strong association of the use of TZD with increased incidence of heart failure, decreased risk of idiopathic cardiomyopathy in patients with type 2 diabetes with TZD use was found in our study. In a meta-analysis of randomized trials [52], the use of pioglitazone leads to a lower risk of death, myocardial infarction, and stroke among a diverse population of patients with type 2 diabetes. TZD, which is an agonist of PPAR-γ, increases insulin sensitivity and glucose uptake in adipose and muscle tissues; suppresses hepatic gluconeogenesis; and diminishes fasting glucose, glycosylated hemoglobin, and plasma insulin levels [53]. Therefore, TZD might have played a role and protection of idiopathic cardiomyopathy in patients with diabetes.

Currently, no long-term studies have assessed the effect of meglitinides on cardiovascular outcomes or mortality in patients with type 2 diabetes. However, a Danish nationwide registry-based observational analysis [54] showed that mortality and cardiovascular risk associated with the use of repaglinide are similar to those of metformin. The reduced risk of idiopathic cardiomyopathy in patients with diabetes using meglitinides deserves further investigations.

Previous cardiovascular outcomes trials showed negative [55, 56] or neutral [57, 58] effects of some DPP4i, but our study showed reduced risks of idiopathic cardiomyopathy in patients with type 2 diabetes who were taking DPP4i. Besides their positive effect on glucose control, DPP-4i has also shown neutral to modest beneficial effects on body weight, blood pressure, postprandial lipemia, inflammatory markers, oxidative stress, and endothelial function in patients with type 2 diabetes [59], which might have conferred favorable effect on cardiovascular outcomes. Further randomized controlled trials are necessary to verify our study results.

The beneficial cardiovascular outcome studies of GLP-1 [60] and SGLT-2i [61, 62] were carried out in very high-risk populations to increase the hazard rate for major cardiovascular events, but the information of putative advantage in lower-risk patients is scarce. In our study, the participants with cardiovascular risk factors had been eliminated before the index date; the risk of cardiovascular outcomes might have been relatively low compared with those in previous randomized studies. We observed that GLP-1 and SGLT-2i remarkably reduced the risk of idiopathic cardiomyopathy in Taiwan’s diabetic population. In the CVD-REAL study, in which data were collected via medical claims, primary care/hospital records, and national registries from the United States, Norway, Denmark, Sweden, Germany, and the United Kingdom, revealed that newly initiated treatment with SGLT-2i is associated with a lower risk of hospitalization for heart failure and death than other glucose-lowering drugs [63]. GLP-1 and SGLT-2i seem to provide cardiovascular benefits to patients with diabetes around the world. The administration of GLP-1 in patients with type 2 diabetes reduces early left ventricular diastolic filling and left ventricular filling pressure and therefore slows the progression of diabetic cardiomyopathy [64]. Adequate glucose loading as energy substrate in GLP-1 therapy prevented heart failure deterioration [65]. Similarly, the mitigation of glycemia-related cardiotoxicity, natriuretic actions and a shift in myocardial fuel utilization might be attributed to the cardiovascular benefits of SGLT-2i [66]. However, the percentage of GLP-1 and SGLT-2i use occupied only a small proportion in Taiwan; hence, further long-term studies that will investigate the possible pathophysiological relationship of the above medications to the reduced risk of idiopathic cardiomyopathy might resolve this phenomenon.

Our study has several methodological strengths. First, the type 2 diabetes and control groups were retrieved from the NHI database, which is population-based and highly representative; therefore, the possibility of selection biases was small. In addition, the likelihood of the non-response and follow-up loss of the cohort members was little. The attainment of disease information from medical claims rather than self-reports may largely reduce the chance of recall bias. Second, one of the potential advantages of using insurance claim datasets in clinical research is the easy access to the longitudinal records for a large sample of patients from different geographic areas [67]. Third, such a large number of study subjects also made age- and sex-stratified analyses possible without compromising the statistical power. Fourth, adjustment for urbanization status reduced urbanization-related confounding, because the diagnostic procedures of cardiomyopathy can be dependent on medical resources and physicians’ behavior,

In spite of the above strengths, over study has several limitations. First, exclusive reliance on the claim data might result in potential disease misclassification bias in our study. A previous study reported that the accuracy of a single diabetes diagnosis in the NHI claim data was 74.6% [68], but we used at least two diagnoses of type 2 diabetes with the first and last visits > 30 days apart, which might have largely reduced the likelihood of disease misclassification. However, the control group might have included people with new onset or undiagnosed diabetes. Such misclassification bias, however, is likely to be non-differential and tends to underestimate rather overestimate the true relative risks [69]. Second, a number of potential confounders including BMI, duration and treatment regimens of diabetes, smoking, alcohol consumption, other socioeconomic characteristics as well as blood pressure, lipid profile, and blood sugar status, in our study, which might have resulted in residual confounding. However, we adjusted cardiovascular risk factors, diagnoses of obesity and hyperlipidemia, and the use of antihypertensive medications in the analysis and still noted a remarkably increased risk of cardiomyopathy in patients with type 2 diabetes. Third, a certain proportion of idiopathic cardiomyopathy might be related to genetic predisposition, but we could not obtain information of family history from the NHI claims. Fourth, although previous studies showed that blood glucose control level and variability might be associated with cardiovascular outcomes, we were unable to investigate this issue as the laboratory data are not available in the NHI claims. Lastly, the data analyzed in this study were totally based on Chinese ethnicity; thus, the generalizability of the study findings to other ethnic populations should be interpreted with caution.

## Conclusions

This study was the first one to evaluate the incidence of idiopathic cardiomyopathy in patients with diabetes based on different sex and the associated risk of various antidiabetic medications used. After a maximum of 10 years of follow-up, except in those men aged > 64 years, the men and women with type 2 diabetes were observed to have increased risk of idiopathic cardiomyopathy by 50% and 80%, respectively, even after the adjustment of underlying cardiovascular risk factors. The patients who resided in rural areas had minimally higher absolute and relative risk of idiopathic cardiomyopathy, especially in female patients. Although the usage of ACEi, ARB, and β-blocker could not reduce the risks of idiopathic cardiomyopathy, the patients taking metformin, meglitinides, TZD, DPP4i, SGLT-2i, and GLP-1 had substantially lower risks of suffering from idiopathic cardiomyopathy. This novel information might be crucial for diabetologists to select optimal therapy for patients with diabetes in daily clinical practice. Diabetic cardiomyopathy has potentially serious medical and economic outcomes. Therefore, this study suggested a need to implement the multifaceted interventional program with particular focus on younger patients with type 2 diabetes.

## Supplementary information

**Additional file 1: Table S1. **Relative hazards of idiopathic cardiomyopathy (ICD9 = 425.4; ICD10 = I42.0) in relation to diabetic and control groups accompanied by selected clinical comorbidities.

## Data Availability

The data sets analyzed during the current study are not publicly available because of information governance restrictions.

## Abbreviations

ACEi: Angiotensin converting enzyme inhibitors
AGi: α-Glucosidase inhibitors
ARB: Angiotensin receptor blockers
CI: Confidence Intervals
DPP-4i: Dipeptidyl peptidase 4 inhibitors
GLP-1: Glucagon-like peptide 1 receptor agonists
HR: Hazard Ratios
ICD-9-CM: International Classifications of Diseases, Ninth Revision Clinical Modification
ICD-10-CM: International Classifications of Diseases, Tenth Revision Clinical Modification
NHI: National Health Insurance
SGLT-2i: Sodium glucose cotransporter 2 inhibitors
SU: Sulphonylureas
TZD: Thiazolidinediones
