# Supplementary material for: Incidence of idiopathic cardiomyopathy in patients with type 2 diabetes in Taiwan: age, sex, and urbanization status-stratified analysis
Source: Cardiovasc Diabetol. 2020 Oct 14;19:177. doi: 10.1186/s12933-020-01144-y (PMC7558694; doi:10.1186/s12933-020-01144-y)
Supplement: Supplementary file 1 — Additional file 1: Table S1. Relative hazards of idiopathic cardiomyopathy (ICD9 = 425.4; ICD10 = I42.0) in relation to diabetic and control groups accompanied by selected clinical comorbidities. [file 12933_2020_1144_MOESM1_ESM.docx]

Table S1. Relative hazards of idiopathic cardiomyopathy (ICD9=425.4; ICD10=I42.0) in relation to diabetic and control groups accompanied by selected clinical comorbidities.

|  | Crude HR^a^ | Adjusted HR^a, b^ |
| --- | --- | --- |
| Control without any risk factors | 1.0 | 1.0 |
| Control with ischemic heart disease | 13.65(11.10-16.80) | 9.22(7.17-11.84) |
| Control with hypertensive disease | 4.93(4.00-6.07) | 3.27(2.49-4.30) |
| Control with rheumatic heart disease | 36.77(27.71-48.79) | 16.14(9.09-28.65) |
| Control with valvular heart disease | 20.78(16.49-26.19) | 12.37(8.87-17.25) |
| Control with congenital heart disease | 17.17(9.49-31.07) | 3.58(1.00-12.81) |
| Control with acute myocarditis | 163.61(67.79-394.89) | 20.31(4.85-85.12) |
| Control with stroke | 4.68(3.65-6.01) | 2.41(1.65-3.51) |
| Control with obesity | 2.90(1.19-7.11) | 0.51(0.13-2.10) |
| Control with hyperlipidemia | 2.69(2.14-3.40) | 1.62(1.19-2.21) |
|  |  |  |
| Type 2 diabetes without any risk factors | 1.0 | 1.0 |
| Type 2 diabetes with ischemic heart disease | 6.98(5.38-9.05) | 5.41(4.05-7.23) |
| Type 2 diabetes with hypertensive disease | 3.18(2.46-4.12) | 2.36(1.76-3.15) |
| Type 2 diabetes with rheumatic heart disease | 16.71(12.21-22.86) | 7.98(4.54-14.01) |
| Type 2 diabetes with valvular heart disease | 12.50(9.47-16.49) | 10.01(6.95-14.42) |
| Type 2 diabetes with congenital heart disease | 10.79(6.74-17.27) | 4.40(2.18-8.88) |
| Type 2 diabetes with acute myocarditis | 30.30(11.17-82.20) | 9.11(2.84-29.23) |
| Type 2 diabetes with obesity | 2.41(1.62-3.59) | 1.29(0.70-2.40) |
| Type 2 diabetes with stroke | 2.88(2.20-3.77) | 1.78(1.27-2.51) |
| Type 2 diabetes with hyperlipidemia | 2.43(1.87-3.14) | 1.68(1.28-2.22) |

^a^ HR= hazard ratio.

^b^ Based on Cox proportional hazard regression with adjustment for age, sex, urbanization status, and antihypertensive medications use.
